# Alterations of the Motor and Olfactory Functions Related to Parkinson’s Disease in Transgenic Mice With a VMAT2-Deficiency in Dopaminergic Neurons

**DOI:** 10.3389/fnins.2020.00356

**Published:** 2020-04-28

**Authors:** Song Jiang, Stefan Berger, Yajuan Hu, Dusan Bartsch, Yanghua Tian

**Affiliations:** ^1^Department of Neurology, First Affiliated Hospital of Anhui Medical University, Hefei, China; ^2^Department of Molecular Biology, Central Institute of Mental Health, Heidelberg University Faculty of Medicine in Mannheim, Mannheim, Germany; ^3^Anhui Province Key Laboratory of Cognition and Neuropsychiatric Disorders, Anhui Medical University, Hefei, China; ^4^Collaborative Innovation Center of Neuropsychiatric Disorders and Mental Health, Hefei, China

**Keywords:** Parkinson’s disease, vesicular monoamine transporter 2, behavioral research, motor disorders, non-motor disorders, mice

## Abstract

Parkinson’s disease (PD) is one of the most common neurodegenerative diseases, with approximately six million people affected worldwide. Vesicular monoamine transporter 2 (VMAT2) dysfunction has recently become a hot topic in the pathophysiology of PD, and the advent of transgenic mice has also accelerated the development of behavioral studies in animal models. However, there are only a few systematic behavioral tests that embrace abundant motor and non-motor performance in a unique mutant mouse model which correspond to the varied symptoms observed in human PD. The aim of this study is to evaluate the responsibility of the unique reduction of dopamine in the varied motor and non-motor symptoms of PD via a transgenic mice model. We analyzed neurotransmitter concentrations in the brain tissue of 18-month-old mutant mice, with selective inactivation of one allele of *Vmat2* in dopaminergic neurons (VMAT2^DATcre^-HET) to confirm the selective reduction of dopamine, and then examined behavioral functions. Neurochemical tests showed lower dopamine concentrations in specific brain regions of VMAT2^DATcre^-HET mice, especially the ventral tegmental area/substantia nigra and striatum, together with relatively unchanging concentrations of norepinephrine and serotonin, demonstrating the dopaminergic specificity of this mouse model. Behavioral tasks showed impairments in several motor functions and major defects in olfactory abilities in the VMAT2^DATcre^-HET mice. However, no significant changes were found in the majority of non-motor tests, such as emotional performance and sleep patterns. We concluded from this study that the selective inactivation of one allele of the *Vmat2* gene in dopaminergic neurons was related to dopamine reduction, resulting in phenotypes resembling some of the major deficits in PD, especially those of motor symptoms and olfactory functions.

## Introduction

Parkinson’s disease (PD) is a common and complicated neurodegenerative disease with motor deficits, including static tremor, rigidity, bradykinesia, and non-motor dysfunctions, such as hyposmia, sleep disturbance, and emotional disorders (Khoo et al., 2013; Kalia and Lang, 2015). It has been proposed that vesicular monoamine transporter (VMAT) dysfunction and the resulting disruption of the cytosolic environment of neurons may play a crucial role in the pathogenesis of PD (Miller et al., 2011; Loho et al., 2019).

Vesicular monoamine transporter 2, encoded by the gene *SLC18A2*, is a subtype of VMAT that is located in the central nervous system (Eiden et al., 2004; Lawal and Krantz, 2013) and is responsible for packaging and transporting monoamines (dopamine, norepinephrine, and serotonin) in small synaptic vesicles in monoaminergic neurons (Bernstein et al., 2014; Lohr et al., 2017). Among all these neurotransmitters, it is generally accepted in the scientific community that dopamine is closely involved in the pathophysiology of PD. In the extracellular space, dopamine performs its physiological function, while accumulation in the extravesicular cytoplasm leads to mitochondrial dysfunction and the formation or stabilization of neurotoxic proteins and oxidative stress (Segura-Aguilar et al., 2014; Arreola et al., 2016; Juárez Olguín et al., 2016). VMAT2 prevents neuron damage via the efficient transport of dopamine. It has been deduced that VMAT2 dysfunction can lead to a reduction of dopamine in vesicles and further intracellular accumulation of dopamine-generated toxic products, ultimately resulting in the degeneration of dopaminergic neurons, particularly in the striatum, which is responsible for PD symptoms (Olanow and Tatton, 1999; Pifl et al., 2014).

A successful animal model and appropriate behavioral assessments could support a better understanding of the critical relationship between VMAT2 and PD and reveal underexplored pharmacological targets (Jagmag et al., 2016). In recent decades, several mouse models have been developed. Although complete deletion of *Vmat2* is lethal (Wang et al., 1997), mice with up to 95% reduction of *Vmat2* can survive to adulthood, although they display a series of dysfunctions caused by progressive nigrostriatal neurodegeneration and the death of dopaminergic neurons (Mooslehner et al., 2001; Caudle et al., 2007). Some further studies also describe different monoaminergic dysfunctions on the VMAT2 hypomorphic mice model, such as the exhibition of progressive noradrenergic neurodegeneration in the locus ceruleus as it preceded degeneration of the substantia nigra pars compacta (Taylor et al., 2014), the disruption of serotonin signaling without degeneration of serotonin neurons (Alter et al., 2016), and especially the general loss of monoamines with some of the non-motor symptoms associated with PD (Taylor et al., 2009). In contrast, mice with overexpression of *Vmat2* have demonstrated neuroprotective effects, with lower toxicity of intracellular toxic wastes (Lohr et al., 2014, 2016).

The previous mouse model studies mentioned above suggested that VMAT2 is associated with not only the pathogenesis of PD but also the potential benefits of upregulating vesicular storage for PD therapy. The studies in Taylor’s lab in particular gave us great inspiration, and they indicated that VMAT2 dysfunction was related to changes of dopamine, norepinephrine, and serotonin, which led to PD symptoms, such as olfactory discrimination and emotional disorders. However, since the key marker and treatment target of PD is dopamine, we plan to focus on the pure dopamine alterations and systematically test the symptoms of PD that fully cover not only the former mentioned non-motor disorders but also motor deficits, which are more typical and considered as core features of PD in clinics. The aim of this study is to evaluate the responsibility of the unique reduction of dopamine in the varied motor and non-motor symptoms of PD via the transgenic mice model. We focused on heterozygous (HET) VMAT2-deficient (VMAT2DATcre-HET) mice that exhibit a 50% reduction in *Vmat2* expression in dopaminergic neurons, and behavioral tasks were used to determine which motor and/or non-motor symptoms related to PD were observed in this mouse model.

## Materials and Methods

### Animals

Mice carrying CreERT2 (Cre recombinase and mutated estrogen receptor binding domain) under the control of the promoter for the dopaminergic-specific dopamine transporter (DAT) gene were crossed with mice carrying the floxed *Vmat2* gene to obtain the target mice, which were maintained on a C57BL/6N background. With the introduction of tamoxifen, one allele of the *Vmat2* gene in the mouse line with DAT-Cre would be conditionally knocked down in dopaminergic neurons, generating VMAT2^DATcre^-HET mice. The rest of the mice with one allele of the *Vmat2* gene were flanked by two loxP sites (VMAT2^flox/+^ mice) and used as the wild-type (WT) mice. The genotypes of all mice were confirmed by PCR with DNA taken from tail samples. The generation and production of the genetic mice were conducted in the Molecular Biology Department of the Central Institute of Mental Health, belonging to the Faculty of Medicine Mannheim of the Heidelberg University.

Two weeks prior to the behavioral tests, the mice were individually housed in standard Makrolon^®^ cages with food and water *ad libitum*, and then groups were transported to a standard animal room (room temperature: 22 ± 1°C; 60% relative humidity) and exposed to a reversed 12-h light/dark cycle (lights off: 7:30 a.m.–7:30 p.m.) to reform the active phase. All procedures involving animals were approved by the regional board of Karlsruhe and carried out in accordance with the German law for the protection of animals with the ethical proposals of the International Association for the Study of Pain (Zimmermann, 1983).

### Neurotransmitter Analyses by HPLC-ED

An independent group of mice, including both WT (VMAT2^flox/+^ mice) and HET (VMAT2^DATcre^-HET mice) mice, was used for neurotransmitter analyses. Tissues from distinct brain regions, including the prefrontal cortex (PFC), ventral tegmental area/substantia nigra (VTA/SN), raphe, hippocampus, nucleus accumbens (NAC), and striatum, of both WT and HET mice (*n* ≥ 6 in each group) were isolated with punching needles in a cryostat (Leica CM3050s, Germany). High-pressure liquid chromatography with electrochemical detection (HPLC-ED) was used to analyze changes in the concentrations of monoaminergic neurotransmitters (dopamine, norepinephrine, and serotonin) and their metabolites, including dihydroxyphenyl acetic acid (DOPAC), homovanillic acid (HVA), and 5-hydroxyindolacetic acid (5-HIAA), in the isolated brain tissues. Data were recorded as concentrations (pmol/mg tissue) or as the DA: DOPAC and DA: HVA ratios.

### Behavioral Testing

All experiments were carried out during the lights-off phase and were conducted with both WT and VMAT2^DATcre^-HET male mice (to avoid hormone disturbances that occur in female mice) (*n* = 10∼14 in each group). The experiments were conducted with increasing stress. Prior to each test, the mice were habituated to the environment for at least 30 min.

#### Open Field Test (in the Dark)

The open field test (in the dark) was conducted to assess locomotor ability (Caudle et al., 2007). The experiment was performed in the dark, recorded by a video camera (Sony CCD IRIS), and analyzed by Etho Vision 3.0 (Noldus Information Technology, Wageningen, the Netherlands). Each mouse was placed individually in the center of the open field apparatus (50 cm × 50 cm × 50 cm, width × length × height) and allowed to explore the field freely for 30 min per day for 2 consecutive days. Movement time, distance moved, speed, vertical-plane entries, and vertical-plane time were recorded and analyzed.

#### Grip Strength

The muscle strength of the mice was evaluated by an automatic force gauge with a triangular grasping bar (Bioseb, France) (Ge et al., 2016). The mouse was placed horizontally toward the base bar of the triangle, allowed to grip it, and then pulled backward by the tail until its grip was lost. The peak force of the forelimbs immediately before grip loss was recorded by the automatic force gauge. Each mouse underwent six trials, and the average data were calculated.

#### Beam Walking

The motor coordination of the mice was tested with a series of narrow beams of different shapes and sizes, which were 80 cm long and elevated 30 cm above the ground (Mooslehner et al., 2001). The mice were given 4 days of training on a 15 mm (width) square beam, with the home cage at the end of the beam as an attractant. On day 5, each mouse performed two consecutive trials on different beams in the following order: 15 mm (width) square, 10 mm (width) square, 15 mm (diameter) round, and 10 mm (diameter) round. The time required to cross each beam was recorded.

#### Footprint Analysis

The walking patterns of the mice were measured with paper, paint brushes, and dye (Marabu GmbH & Co. KG, Germany) for printing forepaws (orange) and hindpaws (blue) (Tillerson et al., 2002). The mouse was trained to traverse a narrow and straight piece of paper (10 cm × 42 cm, width × length) with its home cage at the end of the paper as an attractant. In the test, with the dye on its paws, a track of footprints was left on the recording paper, and stride length (the average distance of each stride on each side) was collected and analyzed.

#### Olfactory Tests

The social and non-social olfactory functions of mice were examined with novel odor exploration tests in a testing box (40 cm × 40 cm × 30 cm, width × length × height) (Taylor et al., 2009). The odor materials were contained in two glass cups (4 cm × 3 cm, diameter × height). At the beginning of each test, the odor materials in the two cups were the same, the mouse was free in the testing box to explore them for 2 min, and the time spent sniffing was recorded. Then the mouse was returned to its cage, and one of the odors was replaced with a novel odor. For the non-social situation, the mouse was expected to distinguish between different natural odors, e.g., odor from bedding scented with cinnamon and odor from normal bedding. In the social odor investigation, the odor-distinguishing materials were the subject mouse’s own cage bedding and bedding from another male mouse’s cage. After the mouse was returned to the testing cage and exposed to the novel odor, the time spent sniffing the odor materials was recorded again.

#### Elevated Plus Maze

An anxiety-like behavioral test was conducted on a cross-shaped maze consisting of two opposing closed polyvinyl arms (8 cm × 40 cm × 10 cm, width × length × height) and two opposing open polyvinyl arms (8 cm × 40 cm × 1 cm, width × length × height; without cover) (Taylor et al., 2009). The maze was elevated 42 cm above the ground. The mouse was first placed in the center of the cross, facing one of the closed arms, and allowed to explore the maze freely for 5 min. The number of times the mouse leaned out to the open arms was recorded.

#### Sucrose Preference Test

The sucrose preference test relied on the natural preference of rodents for sweet nutriment (Fukui et al., 2007). The test was performed over 3 consecutive days, with two bottles containing sterile water or sucrose water at varying concentrations (day 1, 7%; day 2, 0.5%; day 3, 1.5%). The mouse was deprived of fluids for 4 h, and then two bottles were provided for the mouse to make a choice. The sucrose preference ratio was recorded to assess whether the mice were in a depression-like state with anhedonia.

#### Tail Suspension Test

The mouse was suspended by the tail for 5 min, and the immobility time, which reflected the abandonment of active behavior, was recorded by a computer-assisted device (Bioseb, Chaville, France) to assess depression-like behavior (Fukui et al., 2007).

#### Novel Object Recognition Test

The cognitive function of the mice was evaluated in a square open field (40 cm × 40 cm × 30 cm, width × length × height). The mice were exposed to two objects (either glass or plastic) for 5 min; then, the mouse was placed back in its home cage, and one of the objects was changed to a novel object. After 15 min, the mouse was returned to the open field and allowed to explore for another 5 min. The amount of time spent on the original object and the new novel object was recorded separately, and the ratio of time spent exploring the novel object to the total time was calculated (Antunes and Biala, 2012).

#### E-Motion Test

The Mouse E-motion device (Infra-E-Motion, Germany) was placed above the mouse cage for 72 consecutive hours (Taylor et al., 2009). The sleep and activity patterns of mice were monitored, and the data were analyzed to describe the circadian activity of the mice.

### Statistics

The number of mice was 10–14 per group in the behavioral tasks and 6–7 per group in the neurotransmitter analyses. All data were analyzed with SPSS and graphed with Graph-Pad Prism for Windows. The concentrations of monoaminergic neurotransmitters and the behavioral test data were evaluated with Student’s *t*-test or the Mann–Whitney test, depending on whether they were normally distributed. Data are presented as the means ± standard errors of the mean (SEMs). The significance threshold was set at *p* < 0.05, and a trend toward significant results was set at *p* < 0.1.

## Results

### VMAT2^DATcre^-HET Mice Showed a Specific Reduction in Dopamine Concentrations

**FIGURE 1:**
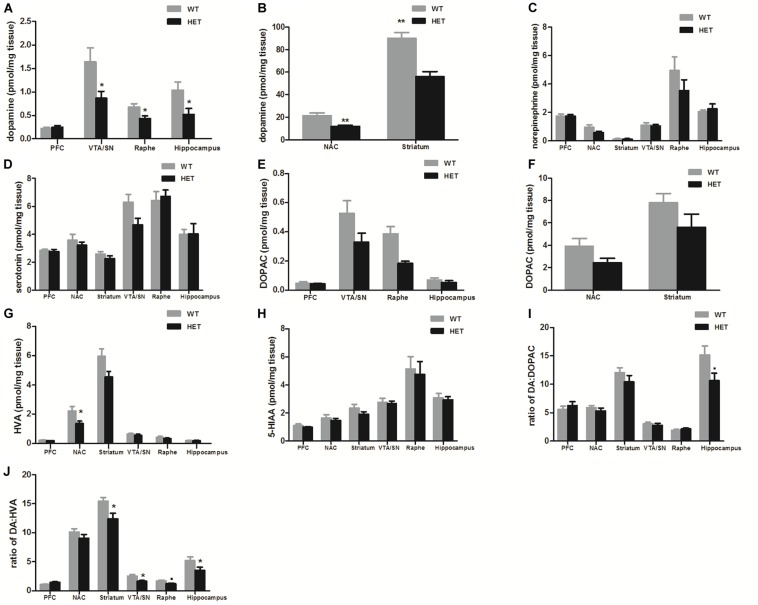
Compared to the WT mice (WT), the VMAT2^DATcre^-HET mice (HET) showed alterations of monoamine concentrations (pmol/mg brain tissue) in different brain regions, with the specific reduction of dopamine concentration in the VTA/SN, raphe and hippocampus **(A)**, and also in the NAC and striatum **(B)**. No significant changes were found on the concentrations of norepinephrine **(C)** and serotonin **(D)**. No significant changes in DOPAC, HVA, 5-HIAAor DA: DOPAC **(E–I)**, but there were significant changes in DA: HVA in the striatum, VTA/SN and hippocampus **(J)**. Data are shown as means ± SEM (*n* = 6–7 animals/genotype; ∙*p* < 0.1;* *p* < 0.05; ** *p* < 0.01).

Compared with the values observed in WT mice, the VMAT2^DATcre^-HET mice showed an ∼50% reduction of dopamine in the VTA/SN (WT, 1.641 ± 0.300 pmol/mg; HET, 0.868 ± 0.146 pmol/mg; *p* = 0.049; Figure 1A), raphe (WT, 0.681 ± 0.070 pmol/mg; HET, 0.433 ± 0.060 pmol/mg; *p* = 0.036; Figure 1A), hippocampus (WT, 1.043 ± 0.170 pmol/mg; HET, 0.524 ± 0.125 pmol/mg; *p* = 0.037; Figure 1A), NAC (WT, 21.526 ± 2.320 pmol/mg; HET, 11.921 ± 1.088 pmol/mg; *p* = 0.005; Figure 1B), and striatum (WT, 90.174 ± 4.923 pmol/mg; HET, 52.938 ± 4.28 pmol/mg; *p* = 0.001; Figure 1B). No significant reduction was detected in the PFC (*p* = 0.682; Figure 1A), and no significant differences in the concentrations of norepinephrine and serotonin were found in the VMAT2^DATcre^-HET mice (Figures 1C,D). In terms of the metabolites of dopamine, there were no significant changes in DOPAC, HVA, 5-HIAA or DA: DOPAC (Figures 1E–I), but there were significant changes in DA: HVA in the striatum (*p* = 0.017; Figure 1J), VTA/SN (*p* = 0.019; Figure 1J), and hippocampus (*p* = 0.041; Figure 1J). These data demonstrated that our mice that were heterozygous for the *Vmat2* allele exhibited a specific reduction of dopamine concentrations in dopaminergic neurons, without alterations of other monoamines, such as norepinephrine and serotonin, which was consistent with our primary model design.

### VMAT2^DATcre^-HET Mice Displayed Motor Dysfunctions

A series of tests related to motor performance were conducted.

**FIGURE 2:**
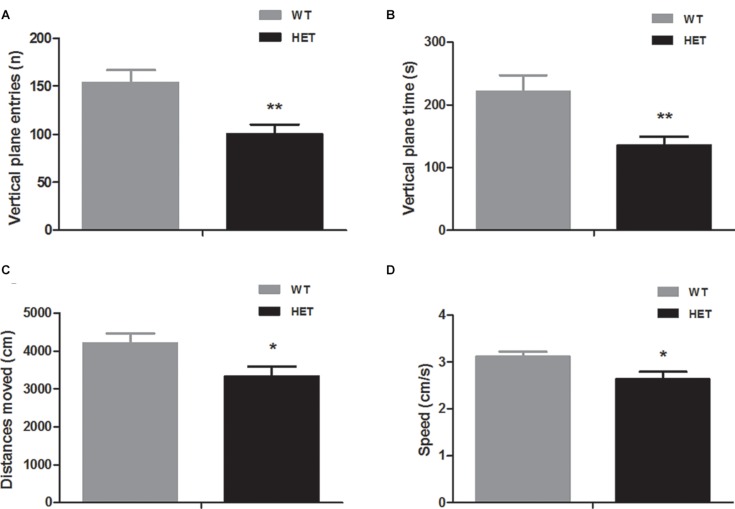
Compared to the WT mice (WT), the VMAT2^DATcre^-HET mice (HET) mice showed worse performances in the open field (in dark) task, indicating worse performances in the vertical-plane exploration, including the number of entries **(A)** and time **(B)** and showing significant disability in the horizontal exploration, including the total distances moved **(C)** and speed **(D)**. Data are shown as means ± SEM (*n* = 10 animals/genotype; * *p* < 0.05, ** *p* < 0.01).

In the open field (in dark) test, compared to the WT mice, the VMAT2^DATcre^-HET mice showed reduced vertical-plane exploration ability, with a trend of fewer vertical-plane entries (WT, 153.80 ± 13.14; HET, 100.30 ± 9.73; *p* = 0.004; Figure 2A) and less vertical-plane time (WT, 221.50 ± 25.36 s; HET, 135.60 ± 13.44 s; *p* = 0.008; Figure 2B). In the horizontal exploration data, the VMAT2^DATcre^-HET mice showed less total distance moved (WT, 4204.70 ± 251.85 cm; HET, 3336.5 ± 251.87 cm; *p* = 0.025; Figure 2C) with lower speed (WT, 3.12 ± 0.10 cm/s; HET, 2.63 ± 0.16 cm/s; *p* = 0.023; Figure 2D).

**FIGURE 3:**
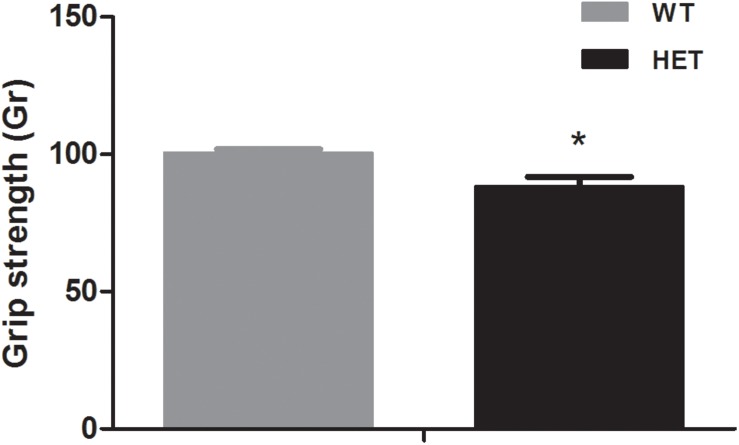
The VMAT2^DATcre^-HET mice (HET) showed weaker grip strength than the WT mice (WT), (average of six trials). Data are shown as means ± SEM (*n* = 10 animals/genotype; * *p* < 0.05).

The grip strength test reflected the muscle condition of the mice. The average grip strength of the WT mice (88.29 ± 3.56 Gr) was weaker than that of the VMAT2^DATcre^-HET mice (100.41 ± 1.41 Gr) (*p* = 0.008; Figure 3).

**FIGURE 4:**
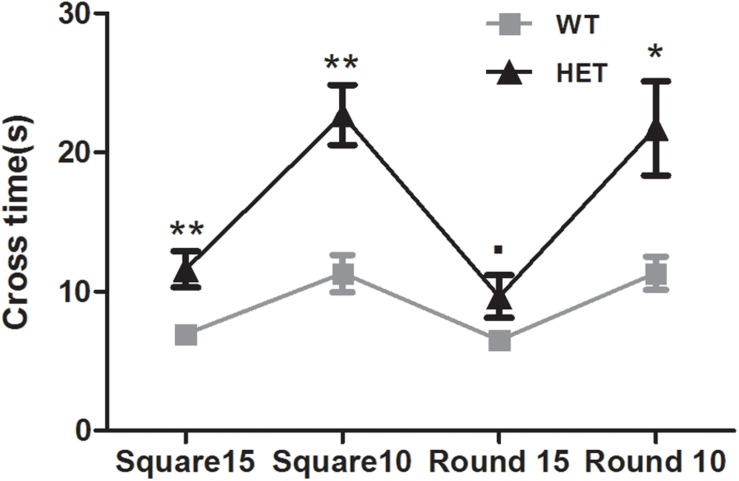
Performances of the VMAT2^DATcre^-HET mice (HET) were worse than the WT mice (WT), on most of the different beams in beam walking test with the longer cross time. Data are shown as means ± SEM (*n* = 10 animals/genotype; ∙ *p* < 0.1; * *p* < 0.05; ** *p* < 0.01).

The beam walking test was used to assess the motor coordination and balance ability of the mice. The VMAT2^DATcre^-HET mice took longer than the WT mice to cross the 15 mm square (WT, 6.95 ± 0.56 s; HET, 11.60 ± 1.28 s; *p* < 0.01; Figure 4), 10 mm square (WT, 11.30 ± 1.33 s; HET, 22.70 ± 2.15 s; *p* < 0.01; Figure 4), and 10 mm round (WT, 11.30 ± 1.20 s; HET, 21.75 ± 3.39 s; *p* < 0.05; Figure 4) beams. The performance on the 15 mm round beam was similar in the two groups (WT, 6.50 ± 0.45 s; HET, 9.65 ± 1.52 s; *p* = 0.063; Figure 4).

**FIGURE 5:**
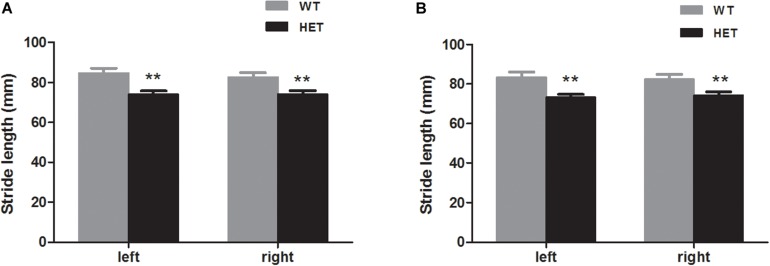
The VMAT2^DATcre^-HET mice (HET) had shorter stride length than the WT mice (WT), both that of the forelimbs **(A)** and the hindlimbs **(B)**. Data are shown as means ± SEM (*n* = 13–14 animals/genotype; ** *p* < 0.01).

Footprint analysis was conducted to record the physical walking patterns of the mice. The VMAT2^DATcre^-HET mice had a shorter stride length than the WT mice for the forelimbs on both sides (WT, left, 84.83 ± 2.31 mm, right, 82.80 ± 2.17 mm; HET, left, 74.03 ± 1.72 mm, right, 74.15 ± 1.74 mm; left, *p* = 0.001, right, *p* = 0.005; Figure 5A) and the hindlimbs on both sides (WT, left, 83.64 ± 2.55 mm, right, 82.35 ± 2.55 mm; HET, left, 73.23 ± 1.46 mm, right, 74.28 ± 1.64 mm; left, *p* = 0.002, right, *p* = 0.015; Figure 5B).

Collectively, these data showed that the reduction of dopamine was associated with significant motor dysfunction.

### VMAT2^DATcre^-HET Mice Were Impaired in Olfactory Function

Hyposmia is a common and early onset symptom of PD. Olfactory tests with different natural or social odors were conducted on the WT mice and VMAT2^DATcre^-HET mice to assess their olfactory function.

**FIGURE 6:**
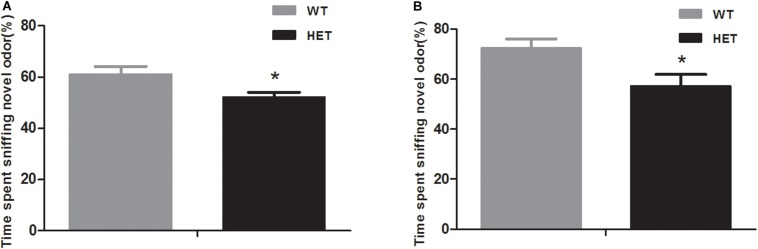
Compared to the WT mice (WT), the VMAT2^DATcre^-HET mice (HET) showed disability in the novel odor exploration tests with both the non-social part **(A)** and the social part **(B)**. Data are shown as means ± SEM (*n* = 14 animals/genotypes; * *p* < 0.05).

In the non-social odor exploration test, the WT mice showed a good ability to distinguish odor; they spent as much as 61.12 ± 3.02% of their time investigating the novel odor material, while the VMAT2^DATcre^-HET mice could not discriminate the odors and spent nearly the same time sniffing the different odor materials (52.21 ± 1.80%, *p* = 0.018; Figure 6A).

In the social odor exploration test, the VMAT2^DATcre^-HET mice showed a weaker ability to discriminate a novel odor, with an investigatory time percentage of 57.18 ± 4.75, compared with the WT mice, which had a percentage of 72.54 ± 3.53 (*p* = 0.021, Figure 6B).

These data demonstrated that reduced dopamine correlated closely with olfactory defects.

### VMAT2^DATcre^-HET Mice Did Not Present Emotional Disorders

Behavioral tasks were performed to determine any anxiety-like or depression-like behaviors in the mice.

**FIGURE 7:**
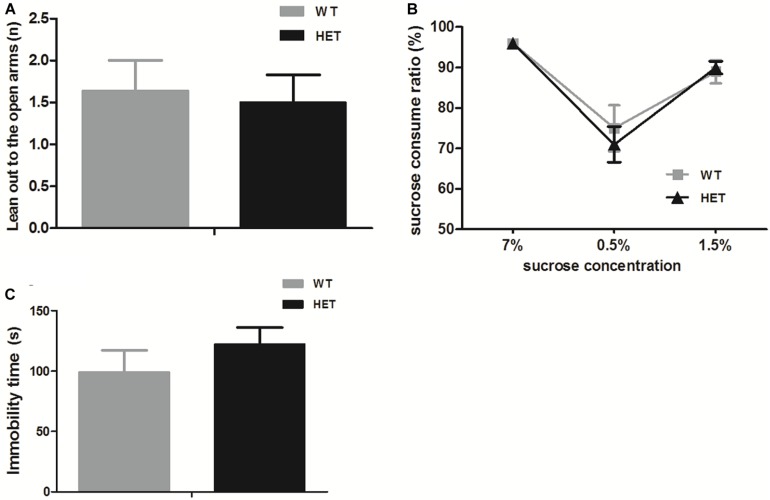
In the emotion-related tasks, both the VMAT2^DATcre^-HET mice (HET) and the WT mice (WT) performed few leaning out to the open arms in the elevated plus mazetest **(A)**, and both of them preferred the sucrose water to the sterile water in the sucrose water test, with a higher sucrose consumption ratio (more than 50%) **(B)**, and struggled in the tail suspension test **(C)**. Data are shown as means ± SEM (*n* = 10–14 animals/genotype).

For anxiety, in the elevated plus maze test, both the WT mice and VMAT2^DATcre^-HET mice preferred to stay in the closed arms, which was reflected by fewer instances of leaning out to the open arms, and no significant genotype difference was found (WT, 1.64 ± 0.36; HET, 1.50 ± 0.33; *p* = 0.77; Figure 7A). For depression, the sucrose preference test relied on the instinct of rodents to prefer sweet nutriment. When sucrose water was given to mice on different days, both the WT and VMAT2^DATcre^-HET mice showed a preference for sucrose water, with nearly the same sucrose preference consumption ratio (compared to water) at different concentrations, and there were no significant differences (Figure 7B). Another additional test for depression-like behavior was the tail suspension test. The immobility time of the WT mice was 99.22 ± 18.14 s, and the VMAT2^DATcre^-HET mice had an immobility time of 122.30 ± 13.87 s, but no significant genotype changes were found (*p* = 0.34, Figure 7C).

These data demonstrated that the elimination of dopamine did not lead to significant emotional disorders.

### VMAT2^DATcre^-HET Mice Did Not Show Significant Disturbances in Cognition or Sleep

**FIGURE 8:**
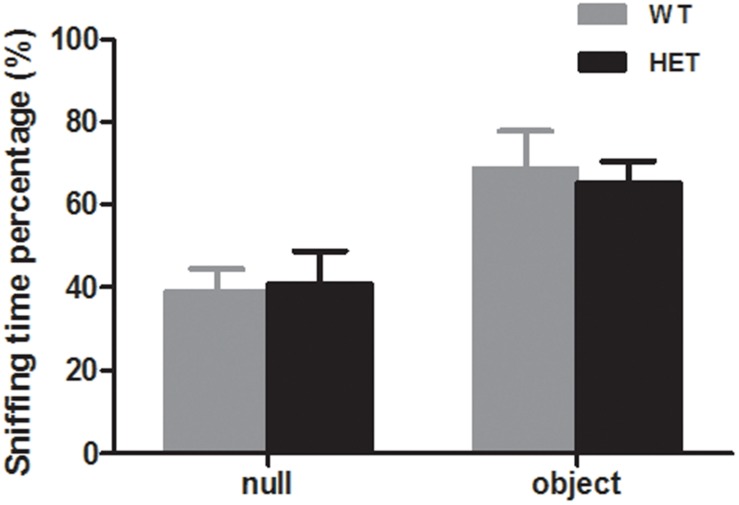
Both the VMAT2^DATcre^-HET mice (HET) and the WT mice (WT) can recognize the familiar subject and show interest in the novel object, with more sniffing time percentage on the novel object. Data are shown as means ± SEM (*n* = 10 animals/genotype).

The novel object recognition test was used to evaluate the cognition of the mice. When exposed to a novel object, both the WT mice and VMAT2^DATcre^-HET mice showed more interest in the novel object (WT, 68.79 ± 9.11%; HET, 65.41 ± 5.16%; *p* = 0.75, Figure 8), and there was no significant difference between the two groups.

**FIGURE 9:**
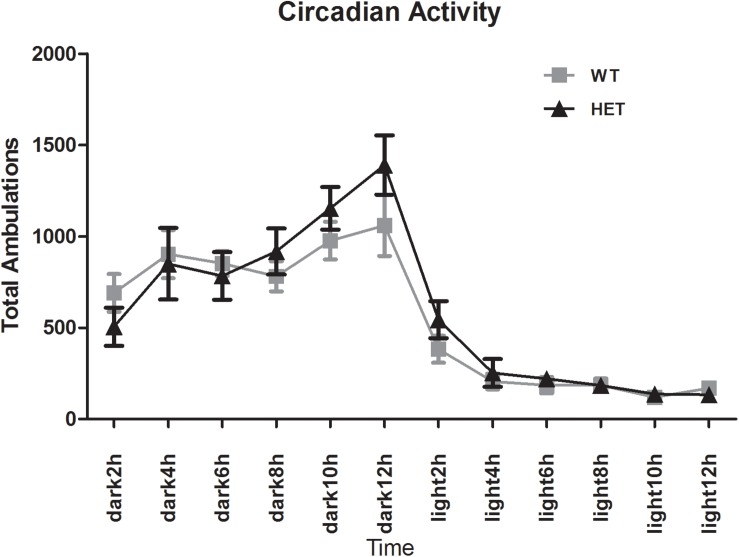
Both the VMAT2^DATcre^-HET mice (HET) and the WT mice (WT) had a clear circadian activity. No sleep disturbance was observed in the VMAT2^DATcre^-HET mice (HET). Data are shown as means ± SEM (*n* = 14 animals/genotype).

The E-motion device showed that both the WT mice and VMAT2^DATcre^-HET mice had clear circadian activity. The mice were active during the dark phase and fell asleep ∼2 h after the light was switched on. No sleep disturbance was observed in the VMAT2^DATcre^-HET mice (Figure 9).

## Discussion

Our study demonstrated that mice with a selective reduction of dopamine concentrations in the brain showed significant impairments in several motor tests and major defects in olfactory abilities but no significant deficits in non-motor functions, such as cognition, emotional performance and sleep patterns.

Compared with the WT mice, the VMAT2^DATcre^-HET mice had lower dopamine concentrations in most regions of the brain, specifically in the VTA/SN and striatum. It has been proposed that reduced dopamine concentrations are related to the inhibition of *Vmat2* expression (Miller et al., 2011). The ablation of the *Vmat2* gene in our mouse line occurred selectively in dopaminergic neurons, and it was expected that other major monoamines, such as serotonin and norepinephrine, would exhibit no significant alterations in the VMAT2^DATcre^-HET mice. In view of the abovementioned facts, it was considered that the VMAT2^DATcre^-HETmouse model was consistent with our primary model design.

Motor behaviors include a range of categories, such as locomotion, muscle condition, and coordination ability. Caudle and associates observed a significant reduction in locomotion in mutant mice expressing ∼5% of normal *Vmat2* levels in the open fields (in the dark) test; this change corresponded to the reduced dopamine concentration in the striatum and could be adjusted by the administration of levodopa (Caudle et al., 2007). Isingrini’s work also showed that the special VMAT2-KO mice specific to the dopamine nigrostriatal pathway moved shorter distances in the open field when 16 weeks post viral injection (Isingrini et al., 2017). Mooslehner subjected mice with a hypomorphic allele of the *Vmat2* gene that expressed a very low level of *Vmat2* to a beam walking test and observed coordination deficits (Mooslehner et al., 2001). In our mouse model, motor symptoms were tested with different tasks. With the open field (in the dark) test, we confirmed that the VMAT2^DATcre^-HET mice showed locomotor activity impairments. With the grip strength test, we found that the VMAT2^DATcre^-HET mice showed a weaker muscle condition, which might be caused by a dysfunction of the link between the cortical and premotor areas related to the basal ganglia (Frazzitta et al., 2015). The performance of mice in the beam walking test was independent of emotional disturbances, as all mice were given sufficient time to habituate and learn to walk on the beams, and as expected, the motor coordination and balance performance of the VMAT2^DATcre^-HET mice were worse than those of the WT mice; this effect appeared to be correlated with the dopamine reduction caused by abnormal vesicle handling. In addition, PD patients typically present with “freezing of gait.” The core feature of this gait is extremely short steps, and patients have difficulty in both starting movements and turning while walking (Nutt et al., 2011). It is thought that muscle stiffness is mainly responsible for the shortened and hurried steps of these patients (Hallett, 2008), and many other theories have also been proposed (Pereira et al., 2016; Mirelman et al., 2019). The footprint analysis test recorded walking patterns. Isingrini’s work showed shorter stride length on the special VMAT2-KO mice specific to the dopamine nigrostriatal pathway when 16 weeks post viral injection (Isingrini et al., 2017).

Taylor and coworkers observed that their Vmat2-deficient mice displayed a shorter stride length at an older age (until 28 months of age), which corresponded to the “shuffling gait” in PD patients (Miller et al., 2011). However, in our study, the VMAT2^DATcre^-HET mice at the age of 18 months presented with a shortened stride length compared to that of the WT mice. It was thus considered that reduced vesicular storage of dopamine caused by VMAT2 dysfunction was associated with motor deficits in PD. All these findings corroborate the suggestion that VMAT2 dysfunction in dopaminergic neurons can be responsible for the motor deficits of mice, which are considered to be essentially dopamine responsive, revealing that reduced intravesicular dopamine is sufficient enough to lead to motor symptoms in PD patients.

According to the theory of Braak, olfactory dysfunction, which is related to the olfactory system, presents in the early stages of the disease, usually before motor symptoms (Braak et al., 2003; Sui et al., 2019). In the clinic, up to 90% of PD patients suffer from hyposmia (Chaudhuri and Schapira, 2006). In a study by Taylor, mice with a 95% genetic reduction of *Vmat2* showed olfactory impairment when distinguishing natural odor from peppermint/vanilla in water at the age of 18 months (Taylor et al., 2009). In our study, VMAT2^DATcre^-HET mice were examined at the same age, and similar olfactory impairments were found. In terms of social odors, Taylor and coworkers found that *Vmat2*-deficient mice showed progressive olfactory deficits from the age of 5–12 months (Taylor et al., 2009). The 18-month-old VMAT2^DATcre^-HET mice in our study also displayed significant olfactory dysfunction. Our olfactory examination confirms that the occurrence of hyposmia preceding motor symptoms is a stable characteristic in PD models and correlates closely with the reduced vesicular storage of dopamine, which is caused by specific VMAT2 dysfunction.

The typical neuropsychiatric non-motor symptoms of PD are depression and anxiety (Balestrino and Martinez-Martin, 2017), and they do not parallel the progression of motor symptoms (Müller et al., 2013; Ehgoetz Martens and Lewis, 2017; Lenka et al., 2017), indicating that they occur through an independent pathway. A core and elusive feature of depression is anhedonia (Coccurello, 2019). For rodents, anhedonia can be characterized by decreased consumption of food, such as sucrose water, and the related test is the sucrose preference test. Fukui and coworkers demonstrated that *Vmat2*-deficient heterozygous mice at the age of 3–5 months showed anhedonia, with weaker responses to 1% and 1.5% sucrose water (Fukui et al., 2007). In addition, Fukui also performed the tail suspension test on *Vmat2* heterozygous mice and observed less struggle activity, which could be enhanced by antidepressants, revealing that the immobility time of the mice was related to the decrease in activity but not to locomotion ability (Fukui et al., 2007). The anxiety-like performance of Vmat2-deficient mice at different ages was tested using the elevated plus maze in Taylor’s laboratory, and it was demonstrated that at an early age of 4–6 months, *Vmat2*-deficient mice spent significantly more time in the closed arms of the maze, which indicated signs of anxiety (Taylor et al., 2009). Most of the previously mentioned studies used a mouse model with complete monoamine loss and reached the general conclusion that depression and anxiety in PD were associated with VMAT2 dysfunction. In our study, we further showed that our VMAT2^DATcre^-HET mice showed specific dopamine loss and did not exhibit significant neuropsychiatric disorders. These abnormalities seem specific for identifying the role of dopamine in neuropsychiatric symptoms, since our mouse model is based on specific VMAT2 loss in dopaminergic neurons. Another study suggested that the emotional changes in PD might be related to a specific loss of dopamine and norepinephrine innervation in the limbic system (Remy et al., 2005). Our results lend support to the idea that not only dopamine but also norepinephrine and serotonin may participate in the pathogenesis of emotional symptoms in PD.

Several other disorders, including cognitive impairment and sleep disruption, are considered to be involved in PD. Approximately 20–50% of patients with PD develop dementia during the long-term disease course, and this represents a great burden for nursing homes (Svenningsson et al., 2012; Majbour and El-Agnaf, 2017). Sleep disruption is also considered to be a very common but complicated symptom and usually appears at an early stage (Garcia-Borreguero et al., 2003). A previous study by Taylor demonstrated that *Vmat2*-deficient mice presented circadian activity similar to that of the controls, although they showed a shorter latency to behavioral signs related to sleep (Taylor et al., 2009). However, similar to other neuropsychiatric functions, no significant dysfunctions of cognition or sleep were found in our dopaminergic-specific VMAT2-deficient mice. In addition, the novel object recognition test could assess the cognitive function, such as the novel recognition and working memory (Antunes and Biala, 2012). According to the Bevins and Besheer’s protocol, the proper interval time between the familiar and the novel stimulus to test short-term memory should be 1 h (Bevins and Besheer, 2006), thus the results in our novel object recognition test may be not related firmly with the working memory of the mice, but could still reflect other cognitive functions, such as the novel recognition ability, which include the processes of detection, attention, and motivation. The VMAT2-deficient mice did not show significant related dysfunctions. In Draoui’s recent study on rats with pharmacological inhibitions of VMAT2 transporters, it was revealed that the short-term memory deficit of PD patients might be related to the 5-HTgic system (Draoui et al., 2019). Data from our study showed that the elimination of dopamine alone did not lead to most of the reported non-motor deficits and implied that all monoamines, not just dopamine, were involved in the emotional, cognitive, and sleep manifestations of PD. Considering the fact that serotonin and norepinephrine share similar structures with dopamine, it is not surprising that they share similar neurotransmitter roles and yield similar neurotoxic effects in neurons (Politis and Niccolini, 2015; Doummar et al., 2018). Although the detailed mechanism underlying the major non-motor manifestations in PD is still unknown, according to our study, the hypothesis that serotonin and norepinephrine, in addition dopamine, contribute to these processes and may even play a dominant role should be considered. Isingrini’s work showed shorter stride length on the special VMAT2-KO mice specific to the dopamine nigrostriatal pathway when 16 weeks post viral injection (Isingrini et al., 2017). The study in Isingrini’s lab also mentioned related mice models designed to study the respective function of monoamines. They tested the VMAT2^DATcre^-HET mice and found significant dysfunction in the open field test. What’s more, the VMAT2 heterozygous mice in the serotonergic neurons with the promoter for the serotonin transporter gene (VMAT2^SERTcre^-HET mice) and the VMAT2 heterozygous mice in the noradrenergic neurons with the promoter for the norepinephrine transporter gene (VMAT2^NETcre^-HET mice) were also tested, but the primary behavioral tasks did not show significant deficits of the motor or non-motor functions (Isingrini et al., 2016). Considering the relationship between VMAT2 and the non-motor symptoms in PD, more research with varied tasks is needed to explore the unsolved problems.

In conclusion, the mouse model and the systematic study method, including behavioral tasks used in our study, highlight the importance of methodology for performing future functional discrimination studies of neurotransmitters and accurately understanding therapeutic findings. In a recent study, it was clarified that treatment with levodopa in combination with carbidopa had no disease-modifying effect on the early stage of PD (Verschuur et al., 2019). Since most of the early onset manifestations are non-motor symptoms (Bridi and Hirth, 2018; Marinus et al., 2018), our study indicates that these dysfunctions may not be closely associated with dopamine, suggesting that supplementation with dopamine may have no beneficial effects on the early stage of PD. In addition, the potential effects of long-term use of dopamine supplements such as levodopa are not yet clear. Another study implied that levodopa accelerated the damage of dopamine nerve terminals in the substantia nigra (Walton-Hadlock et al., 2004). Levodopa increases the dopamine concentration in the synaptic cleft and improves some symptoms. Then, excess plasma dopamine would be transferred into the cell via “reuptake” by membrane DATs, which is balanced with excretion by VMAT2 to prevent cellular damage. It could be proposed that the dysfunction of VMAT2 in PD patients is related to the accumulation of dopamine and its toxic metabolites, resulting in neuronal loss, during long-term levodopa use. Utilizing dopamine neuron-selective VMAT2-deficient mice as a unique model of PD provides a better understanding of the specific functions of neurotransmitters and facilitates the potential adjustment of therapeutic strategies to improve the quality of life of PD patients.

## Data Availability Statement

The datasets generated for this study are available on request to the corresponding author.

## Ethics Statement

The animal study was reviewed and approved by the Committee on Animal Care and Use (Regierungsprasidium Karlsruhe).

## Author Contributions

SJ, DB, and YT designed the experiments. SJ performed the behavioral tests. SJ and SB conducted the neurotransmitter analyses. SJ and YH analyzed the data. SJ wrote the manuscript and YT correct the manuscript.

## Conflict of Interest

The authors declare that the research was conducted in the absence of any commercial or financial relationships that could be construed as a potential conflict of interest.
